# An overview of resistance to chemotherapy in osteosarcoma and future perspectives

**DOI:** 10.20517/cdr.2022.18

**Published:** 2022-06-23

**Authors:** Dorian Yarih Garcia-Ortega, Sara Aileen Cabrera-Nieto, Haydee Sarai Caro-Sánchez, Marlid Cruz-Ramos

**Affiliations:** ^1^Cirugía Oncológica, Instituto Nacional de Cancerología (INCAN), Ciudad de México 14080, México.; ^2^Posgrado en Ciencias Médicas, Facultad de Ciencias de la Salud, Universidad Anáhuac, Ciudad de México 52786, México.; ^3^Departamento de Patología, Instituto Nacional de Cancerología (INCAN), Ciudad de México 14080, México.; ^4^Cátedras de Consejo Nacional de Ciencia y Tecnología (CONACYT), Instituto Nacional de Cancerología, Ciudad de México 14080, México.

**Keywords:** Osteosarcoma, therapy resistance, tyrosine kinase inhibitor, tumoral extracellular microenvironment, cell cycle

## Abstract

Osteosarcoma (OS) is the most common type of bone sarcoma. Despite the availability of multimodal treatment with surgery and chemotherapy, the clinical results remain unsatisfactory. The main reason for the poor outcomes in patients with OS is the development of resistance to methotrexate, cisplatin, doxorubicin, and ifosfamide. Molecular and cellular mechanisms associated with resistance to chemotherapy include DNA repair and cell-cycle alterations, enhanced drug efflux, increased detoxification, resistance to apoptosis, autophagy, tumor extracellular matrix, and angiogenesis. This versatility of cells to generate chemoresistance has motivated the use of anti-angiogenic therapy based on tyrosine kinase inhibitors. This approach has shown that other therapies, along with standard chemotherapy, can improve responses to therapy in patients with OS. Moreover, microRNAs may act as predictors of drug resistance in OS. This review provides insight into the molecular and cellular mechanisms involved in the development of resistance during the treatment of OS and discusses promising novel therapies (e.g., afatinib and palbociclib) for overcoming resistance to chemotherapy in OS.

## INTRODUCTION

Osteosarcoma (OS) is the most common type of bone sarcoma; these tumors form a heterogeneous group of malignant neoplasms characterized by the production of osteoid matrix^[1,2]^. OS is disseminated by the hematologic route, and the lungs are the main site of metastasis^[2-4]^. It is considered that up to 70% of patients with OS have at least one micrometastatic disease at this site at the time of diagnosis^[5]^. The addition of chemotherapy to the treatment of patients with localized disease OS improves their prognosis, increasing their survival rate by up to 70%^[1,4,6]^. However, metastatic patients continue to have a poor prognosis at 20%-30%^[7]^.

The current standard treatment modalities for OS are neoadjuvant chemotherapy, surgery and adjuvant chemotherapy. Chemotherapy is based on different combinations of doxorubicin (DOX), cisplatin, and methotrexate (MTX). The administration of the latter depends on the age of the patient and may be combined with ifosfamide (IFO), etoposide, *etc*.^[8,9]^. The current chemotherapy scheme was first introduced in the late 1970s and remains virtually unchanged despite numerous efforts to improve treatment outcomes^[10]^. One of the current clinical challenges is drug resistance, which may be inherent or acquired^[11]^. Additionally, patients with poor response and refractory disease (i.e., recurrent or progressive) have a bleak prognosis due to the limited number of effective options in second- or third-line chemotherapy^[12-14]^.

This situation has generated several lines of research to identify the pathophysiological mechanisms by which OS cells develop drug resistance and, thus, devise new strategies based on the utilization of biomarkers or targeted therapies^[11,15,16]^. The high degree of heterogeneity observed in OS renders therapy even more problematic; for instance, the discovery of reliable biomarkers, recognition of the mechanism of recurrence, and identification of cell types that cause OS pose challenges to investigators^[17]^. Cancer cells can utilize various mechanisms to circumvent or counteract the cytotoxic stimuli induced by anticancer therapy: (1) impairment of drug transport and enhancement of drug efflux; (2) increase in deoxyribonucleic acid (DNA) repair; (3) alterations in cell cycle and apoptosis; (4) activation of signal transduction pathways; (5) the tumor microenvironment (TME) and angiogenesis; (6) autophagy; (7) micro-RNA; and (8) maintenance of a stem cell-like phenotype^[18]^. The purpose of the present narrative review is to summarize the advances achieved thus far in this setting and present some perspectives for the treatment of OS in the future using novel drug combinations.

## IMPAIRED INTRACELLULAR ACCUMULATION

OS cells decrease drug accumulation to overcome the cytotoxic effects of chemotherapeutic agents. Insufficient drug transport can be related to reduced folate carriers on the cell membrane, increasing drug efflux, or inducing alterations in target enzymes^[18]^.

### Insufficient drug transport

**Figure 1:**
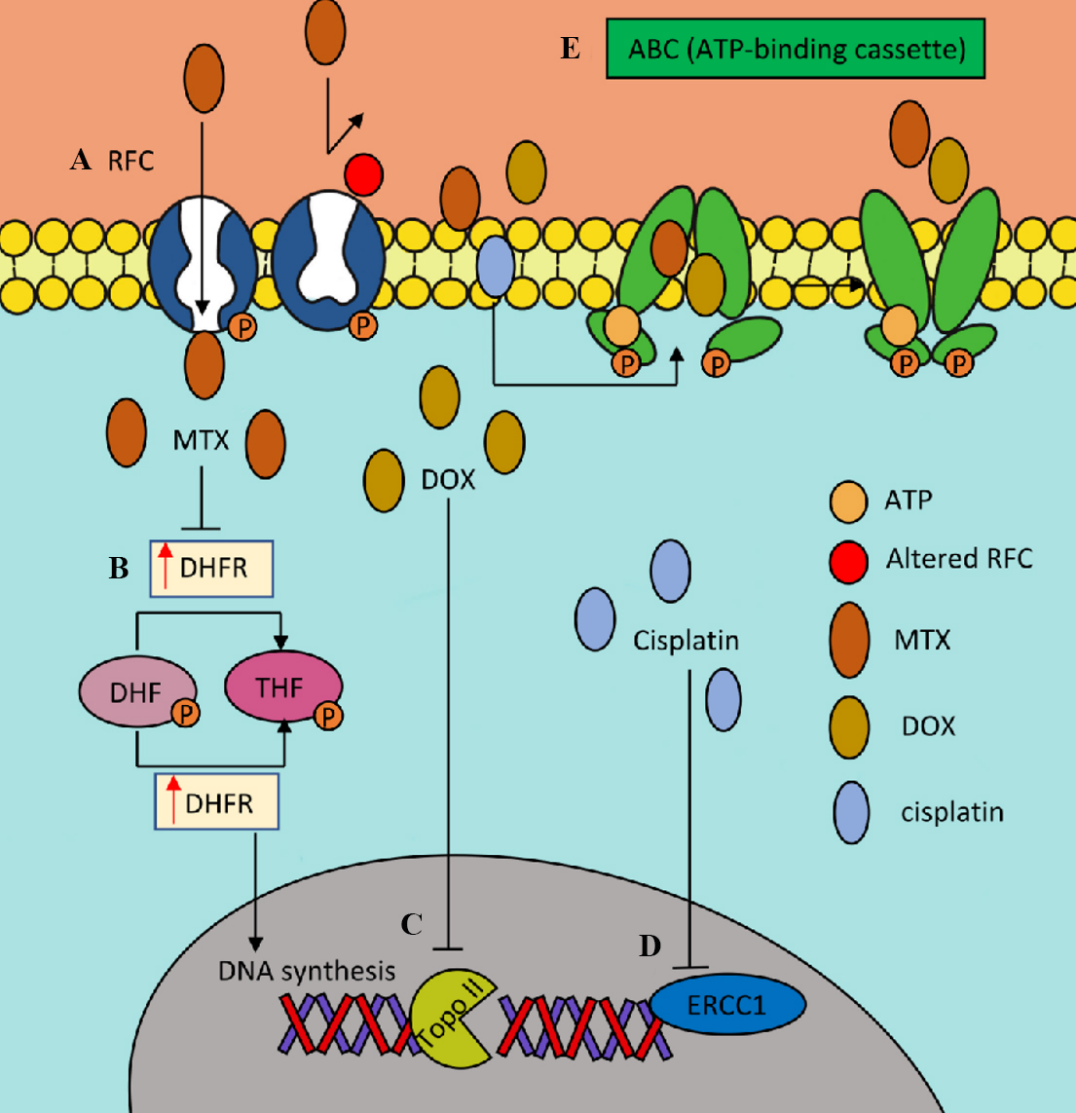
Reduced intracellular drug accumulation. (A) Decreased expression of the reduced folate transporter (RFC) or decreasing transporter function promotes methotrexate resistance. (B) Overexpression of dihydrofolate reductase (DHFR) responsible for the reduction of dihydrofolate (DHF) to tetrahydrofolate (THF) or alterations in the binding affinity of DHFR for methotrexate (MTX) are related with MTX resistance. (C) Decreased expression or mutation of topoisomerase II (Topo II) is associated with doxorubicin (DOX) resistance. (D) High levels of excision repair cross-complement protein 1 (ERCC1) proteins are related to cisplatin resistance. (E) Increased expression of multidrug resistance gene encoding the ATP-binding cassette (ABC) family leads to increased drug efflux and decreased intracellular drug accumulation increasing resistance to DOX and MTX.

Impaired drug transport is a well-described mechanism of resistance to chemotherapy in OS. In particular, this is achieved through a decrease in transporters present on the membrane of tumor cells. MTX is an anti-folate that uses the reduced folate carrier (RFC) to enter OS cells. Following entry, MTX is polyglutamylated to be retained in the cells. Subsequently, MTX inhibits dihydrofolate reductase (DHFR) - the enzyme that converts the dihydrofolate to tetrahydrofolate - which is a one-carbon donor for the de novo synthesis of purine and thymidine. DHFR is essential for the de novo synthesis of DNA, and the interaction between MTX and DHFR prevents DNA synthesis^[18]^. Treatment-resistant OS cells have reduced expression of RFC^[19]^. The decreased expression of RFC in the tumor is associated with the development of resistance to MTX and poor histological responses to preoperative chemotherapy^[20]^. Drugs, such as trimetrexate, do not require RFC for transport. In a phase 2 clinical trial including patients with relapsed OS, toxicity was acceptable, myelosuppression was the major side effect, objective response was 8% (*n* = 39; complete response = 1, partial response = 2, mixed response = 1, and stable disease = 8)^[21-23]^. The combination of trimetrexate with high-dose MTX is currently being tested in a phase 1 trial to evaluate the efficacy, safety profile, and most appropriate dose of trimetrexate (clinical trial identifier: NCT00119301)^[24] ^[Figure 1].

### Enhancement of drug efflux

Drug resistance in OS has been linked to an increase in drug efflux, particularly for DOX. This acquired resistance mechanism is termed multidrug resistance (MDR) and is associated with the overexpression of members of the ATP-binding cassette (ABC) family of efflux transporters. Principally, this process involves the multidrug resistance protein 1 (*MDR1*) gene, which encodes P-glycoprotein (P-gp), also termed MDR1 or ATP-binding cassette subfamily B member 1 (ABCB1). These transporters are active pumps for drugs, such as DOX^[18,25]^. OS cells exhibit high expression levels of ABC transporters, such as MDR1, and are resistant to DOX. An association between MDR1 overexpression and reduced DOX accumulation has been reported. In patients with OS, the overexpression of ABC transporters is associated with poor response to chemotherapeutic agents (e.g., DOX) and worse clinical outcomes^[20,26]^. In preclinical models, the knockout of ABCB1 restored sensitivity to DOX. Drugs, such as trabectedin, may inhibit the transcriptional activation of MDR1; trabectedin modulates gene expression in a promoter manner, affecting the MDR1 gene promoter, thereby emerging as a potential alternative therapeutic strategy for the restoration of sensitivity to DOX^[27]^.

### Alterations in target enzymes

Secondary to alterations in enzymes, increased levels of target enzymes or degreased drug affinity can result in resistance to chemotherapeutic agents. MTX-resistant OS cell lines overexpress DHFR, and high expression of DHFR is also observed in OS metastasis^[28,29]^. DNA topoisomerases II (TOP2) are nuclear enzymes involved in the regulation of DNA topology; TOP2 forms a homodimer that functions by cleaving double-stranded DNA, coiling a second DNA duplex through the gap, and re-binding the strands. TOP2 is essential for cell replication and viability and is recognized as a target of doxorubicin^[30]^. In human cells, two isoforms are described, α and β; in several tumors, low expression of *TOP2B i*s described in DOX resistance, particularly OS DOX-resistant cells had reduced expression of *TOP2B* compared with DOX-sensitive cells^[31]^. Amplification or deletion in TOP2 genes has been described in OS patients. *TOP2B *was deleted in 40.5% of cases and is related to worse event-free survival*. TOP2A* is amplificated in 21% and deleted in 25% of OS tumors. Both gene alterations (amplification and deletion) in *TOP2A *have been correlated with a good response to neoadjuvant chemotherapy and also related to DOX resistance^[32]^. Exploring these enzymes in clinical trials could give us possible utility as biomarkers to predict chemotherapy response.

Several mutations in tumor suppressor genes and other pathways are involved in OS failure to chemotherapy. The most frequent gene altered in this pathology is tumor suppression gene *P53,* and mutations in other cancer drivers such as *RB1, ATRX2, DLG23, RUNX2, WRN, RECQL4, CDKN2A/B, BLM, PTEN,* and PI3K/AKT/mTOR pathway members are described in OS tumors. Some of these genes are involved in cell cycle control and DNA damage repair^[33]^.

## OS AND ALTERATIONS IN DNA REPAIR

It is established that chemotherapeutic agents cause DNA damage that leads to cell death. However, tumor cells can occasionally resist treatment by enhancing their DNA repair pathway. Cisplatin is one of the most studied drugs for which resistance due to an enhanced DNA repair in OS has been reported^[18,34,35]^. Nucleotide excision repair (NER) is an important DNA damage removal pathway. It is implicated in cancer progression and response to platinum-based chemotherapy. Additionally, NER is one of the most studied pathways in the development of drug resistance in OS. NER proteins can repair chemical drug-induced DNA damage. Members of the NER pathway that have been studied thus far include DNA excision repair proteins and excision repair cross-complement proteins (ERCC); these endonucleases are involved in the excision of the lesion, followed by DNA replication and repair process. Defects in NER accumulate DNA damage favoring cancer phenotype. Single nucleotide polymorphism (SNPs) of *ERCC1* and *ERCC2* genes may decrease the expression of *ERCC1 *and *ERCC2 *genes and are associated with chemotherapy response for OS, particularly reduced resistance to cisplatin-based chemotherapy^[36,37]^. In addition, silencing of the *ERCC1*,* ERCC2*,* ERCC3*, and *ERCC4* genes increases sensitivity in resistant OS cell cultures (U2OS/cisplatin 300 and U2OS/cisplatin 1)^[35]^. Base excision repair (BER) is also involved in OS. BER is primarily active in DNA damage caused by small chemical alterations or base loss due to hydrolysis of glycosyl DNA bonds. DNA damage is removed by glycosylases, a complex formed by APEX1 endonuclease, poly(ADP-ribose) polymerase (PARP), and DNA ligase, and XRCC1 recognized the bases sites. Different members of BER are involved in OS; apurinic/apyrimidinic endonuclease (APE-1) has been associated with shorter survival in patients with OS^[18,37]^.

DNA double-strand breaks (DSBs) represent a challenge to genomic integrity. DSBs are detected by a cascade of proteins, involving the process of homologous recombination (HR) or nonhomologous end-join (NHEJ). DSBs activate pathways such as ATM and ATR that result in the phosphorylation of multiple targets, including histone H2AX and checkpoint mediator proteins (CHK) 1 and 2, to finally activate P53. ATR/CHK1 signaling is linked with the activation of BRCA2, recruiting BRCA1 and RAD51, which form filaments on the single-stranded DNA to repair the site of the DNA damage. *BRCA1* and -*2 *mutations lead to impairment to repair DSBs mediated by HR. Deficiency of inhibition of PARP1 in normal cells results in an impairment of the BER response, causing lesions that should be repaired by BER activating HR pathway; however, OS presents mutations in different “BRCA” genes such *PALB2, CHEK2, PTEN,* and *ATM,* resulting in chromosomal instability analogous to *BRCA1/2 *mutations^[33]^, which make it difficult to repair DNA lesions. Exposing BRCA1/2-deficient cells to PARP inhibition results in lethal DNA damage accumulation; consequently, PARP inhibition results in the targeted tumor cell death in BRCA-deficient cancer. Preclinical data suggest the effect of PARP inhibitors in OS MNNG/HOS cells carrying disruptive gain in the *PTEN *gene and deletion of *ATM *gene; the combination of talazoparib, a phase 3 PARP inhibitor, with topoisomerase I inhibitor SN-38 considerably decreases the viability of MNNG/HOS cells, and olaparib was tested in HOS and MG-63 cells with good results. In addition to these preclinical data, it seems that the inhibition of PARP1/2 in *BRCA1/2 *tumor suppressor mutated cells is involved in drug resistance in OS. The use of olaparib (a PARP1 inhibitor) sensitizes OS cell lines to treatment with DOX^[38]^. These types of in vitro studies led to PARP inhibitors being tested in the clinical setting. The ongoing NCT03233204 study investigates the combination of olaparib plus DOX in patients with refractory OS^[39]^. The TOMAS trial tested olaparib and trabectedin in sarcomas. This trial included seven patients with bone cancer; unfortunately, none of the patients showed an objective or clinical response^[40]^, probably due to the small number of patients included in the trial. Furthermore, other combinations, such as olaparib plus ceralasertib (AZD6738), an orally available morpholino-pyrimidine-based inhibitor of ataxia telangiectasia and rad 3-related (ATR) kinase, were tested in this trial (clinical trial identifier: NCT04417062)^[41]^. Recently, the RB pathway has been described as PARP inhibitor (PARPi) sensitive; RB1-defective OS revealed hypersensitivity to the PARPi olaparib, and RB1-defective OS cells may yield BRCAness/HR defects by inducing RAD51 recruitment. Olaparib increases H2AX histone marker and CHK1 phosphorylation^[42]^. RB is highly mutated in OS patients. The MATCH trial is a precision medicine cancer treatment clinical trial where patients are assigned to receive treatment based on their genetic changes. This trial involves children, adolescents, and young adults with advanced cancers, including rare cancers such as osteosarcoma. Olaparib will be tested in patients with defects in DNA damage repair genes and BRCA1/2 mutations. We await the evolution in patients with RB1-mutated disease if they are included^[39,43]^. The potential benefits of anti-PARP treatment will have to wait for the publication of the results of this trial to evaluate future areas of opportunity with this inhibitor.

## CELL CYCLE AND APOPTOSIS DISTURBANCES

DNA damage is one of the principal action mechanisms of chemotherapy, leading to cell death through apoptosis. For their survival, tumor cells arrest their cell cycle to repair DNA damage, thereby evading apoptosis^[44]^. Disturbances in the cell cycle and apoptosis are involved in the development of resistance to chemotherapy in OS cells. For example, following the overexpression of murine double minute 2 (*MDM2*) (a downstream mediator of p53) in tumor cells, p53-mediated apoptosis is inhibited, and cells develop resistance to DNA-damaging agents^[18,45]^. The amplification of *MDM2* is present in 20% of OS cells^[46]^.

### Cyclin-dependent kinase 4

Cyclin-dependent kinase 4 (CDK4) is an enzyme encoded by the *CDK4* gene. The activity of this kinase is restricted to the G1-S phase, and it is responsible for the phosphorylation of the retinoblastoma (RB) protein. The amplification of *CDK4* is found in 20% of OS cells^[46]^. In a study of 50 pediatric and adolescent patients diagnosed with high-grade OS, the copy number analysis detected a recurrent gain of chromosome 12q14.1. This observation was more frequent in the poor responder cohort than in the good responder cohort, where the *CDK4 *gene was associated with copy number gains^[47]^. CDK4 is highly expressed in human OS tissues and cell lines compared with normal human osteoblasts. Elevated CDK4 expression is associated with metastasis and poor prognosis in patients with OS^[48]^. Overexpression of CDK4 is also related to resistance to cisplatin; treatment of U2OS cells overexpressing CDK4 with CDK4/6 inhibitor palbociclib facilitates apoptosis and decreases cell viability in a dose-dependent manner^[47]^. The combination of sorafenib (a multikinase inhibitor) and palbociclib in a cisplatin-resistant, patient-derived, orthotopic, xenograft mouse model of OS resulted in tumor regression and enhanced tumor necrosis^[49]^. Patients with co-amplification of *MDM2* and *CDK4* were treated with the *MDM2* inhibitor ALRN-6924 and palbociclib; the study included ten liposarcomas, one OS, and one glioblastoma. The study concluded that the combination of ALRN-6924 and palbociclib was feasible and well tolerated^[50]^. Palbociclib is currently tested in pediatric tumors, including osteosarcoma, with activated alterations in cell cycle genes in the pediatric MATCH treatment trial (NCT03526250)^[51]^. Abemaciclib is an inhibitor of CDK4/6; Wang *et al.* evaluated the efficacy of abemaciclib in OS cells and an animal model^[52]^. Abemaciclib inhibited growth and anchorage-independent colony formation of OS cells and inhibited tumor formation and growth in a dose-dependent manner in the animal model. Abemaciclib combined with DOX results in much greater efficacy than DOX alone in inhibiting tumor growth; it acts by suppressing the CD4/6-Cyclin D-Rb pathway. A clinical trial with OS and abemaciclib is ongoing (NCT04040205)^[52,53]^.

### Myeloid cell leukemia-1 protein and its potential role in OS

Cell death signaling is orchestrated by members of the B cell lymphoma 2 (BCL2) family that contains antiapoptotic proteins (e.g., BCL2 and BCLXL) and proapoptotic proteins [e.g., BCL2 associated X (BAX)]. Inhibition of BCL1/BCLXL enhanced the chemosensitivity of OS to DOX and cisplatin. Myeloid cell leukemia-1 (MCL-1) is a pro-survival member of the BCL2 family, contributing to the avoidance of cell death by acting as a regulator of apoptosis in some human malignancies^[54]^. In OS, MCL-1 expression is upregulated after chemotherapy, and high MCL-1 expression is associated with poor overall survival, increased recurrence rate, decreased sensitivity to MTX, and promotion of tumor proliferation^[55]^. In OS cells, MCL-1 is a direct target of miR-375; overexpression of miR-375 enhances the effects of cisplatin-induced DNA damage mediated by MCL-1^[54]^. Regorafenib is an oral type II multikinase inhibitor that inhibits the vascular endothelial growth factor receptor 1-3 (VEGFR1-3), platelet-derived growth factor receptors, fibroblast growth factor receptors (FGFR), tyrosine kinase receptor with immunoglobulin-like and EGFR-like domains 2 (TIE-2), and pathways involved in angiogenic and metastasis process. Sorafenib has been approved as a second-line treatment in OS, as discussed below^[56]^. MCL-1 is an essential survival factor for endothelial cells (EC) required for blood vessel production during angiogenesis. Deletion of MCL-1 in EC cells resulted in a dose-dependent increase in EC apoptosis in the angiogenic vasculature and reduced vessel density. Inhibition of vascular endothelial growth factor A (VEGF-A) may cause EC apoptosis^[57]^. The role of MCL-1 in regorafenib resistance has been evaluated in colorectal cancer. Regorafenib-resistant cells are deficient in MCL-1 degradation, MCL-1 is associated with PUMA and inhibits apoptosis, and MCL-1 inhibitor overcomes acquired resistance to regorafenib by liberating PUMA from MCL-1, restoring apoptosis. Thus, inhibition of MCL-1 also seems to overcome the resistance to regorafenib^[58]^.

MCL-1 appears to be a new therapeutic target in OS. Inhibitors of MCL-1 can be used in different settings, e.g., to restore sensitivity to chemotherapy or anti-angiogenic resistance or in combination with other agents to increase therapeutic efficacy. A phase 1 study of a MCL-1 inhibitor in solid tumors, including sarcomas, is currently ongoing^[59]^.

## SIGNAL TRANSDUCTION PATHWAYS

### Human epidermal growth factor receptor family and OS

**Figure 2:**
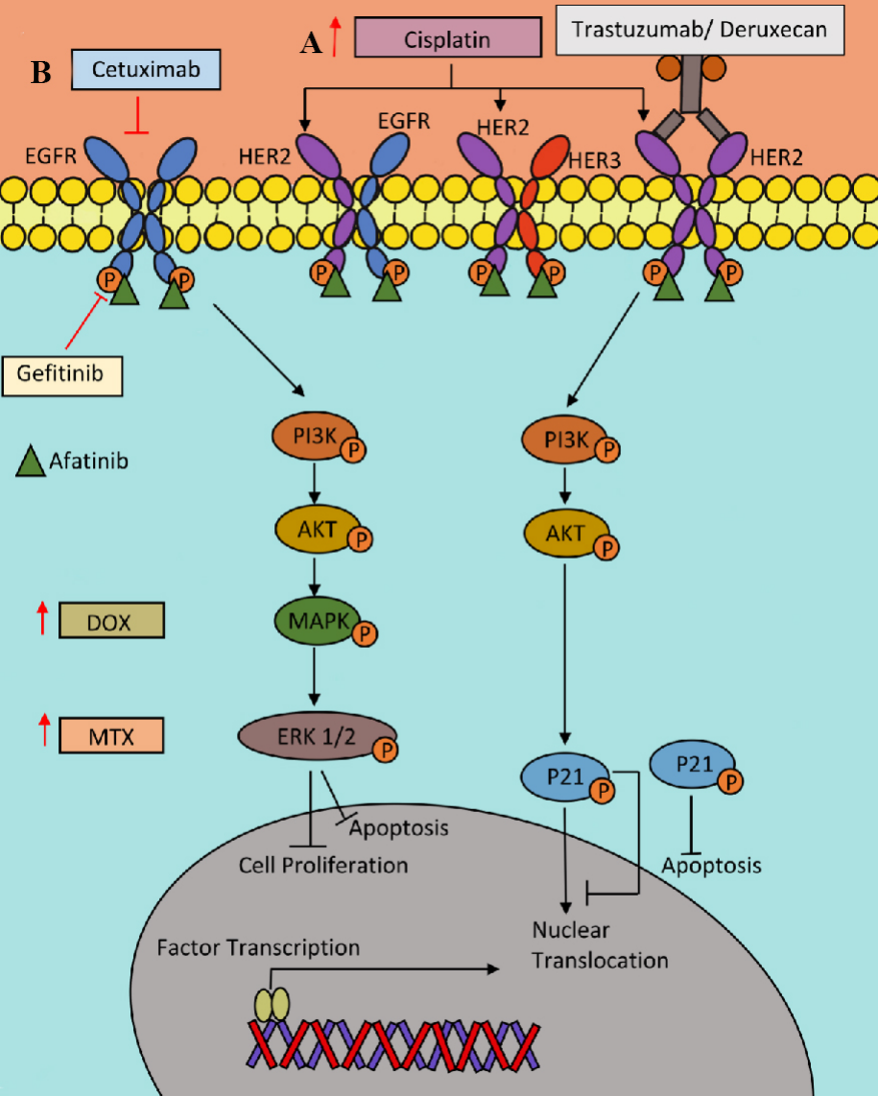
Role of Family HER Pathway in osteosarcoma chemotherapy resistance. (A) Cisplatin resistance is associated with HER2 overexpression, PI3K/AKT activation and promotion p21 nuclear exclusion, favoring cell cycle arrest and proliferation. (B) Anti-EGFR therapy such as cetuximab and gefitinib sensitized osteosarcoma cells to DOX and MTX. (C) Afatinib a pan-HER Family inhibitor have an inhibition effect in osteosarcoma cell proliferation, migration an invasion. (D) Trastuzumab deruxtecan is an antibody-drug conjugated composed by anti-HER2 humanized monoclonal antibody and a topoisomerase I inhibitor as cytotoxic drug, that is now been tasted in clinical trials. AKT: Protein kinase B; DOX: doxorubicin; EGFR: epidermal growth factor receptor; ERK: extracellular signal-regulated kinase; HER: human epidermal growth factor; MTX: methotrexate; PI3K/AKT: phosphoinositide 3-kinase.

Since the 1990s, the expression of human epidermal growth factor receptor 2 (HER2) has been reported in OS primary tumors and metastases^[60]^. HER2 is overexpressed in approximately 32%-45% of OS samples. Some studies have associated HER2 with worse event-free survival and metastasis-free survival; they have also correlated the overexpression of HER2 with poor response to chemotherapy^[60]^. Based on this evidence, strategies directed against HER2 to increase the survival of patients with OS HER-positive tumors have emerged. The use of trastuzumab in combination with cytotoxic chemotherapy was investigated in a phase 2 trial of 96 patients newly diagnosed with metastatic OS; of those, 41 had tumors expressing HER2. There was no difference in event-free survival or overall survival between the HER2-positive and HER-negative groups; trastuzumab has not been tested in other randomized trials^[61]^. The location of HER2 in OS probably contributes to the failure of treatment with monoclonal antibodies; however, there is a need to develop other intracellular inhibitors for the blockage of this pathway. Overexpression of HER2 has been involved in mechanisms of resistance to cisplatin mediated by phosphoinositide 3-kinase/protein kinase B (PI3K/AKT) activation. The basal activity of PI3K/AKT1 upregulates cyclin-dependent kinase inhibitor 1A (CDKN1A; also termed p21), promoting cell cycle arrest and leading to time for DNA repair; additionally, HER2 overexpression mediates the nuclear exclusion of p21 and activation of PI3K/AKT, favoring cell proliferation. Both aforementioned mechanisms have been described in resistance to cisplatin^[34,62]^. In addition, overexpression of HER2 has been associated with resistance to cisplatin in clinical settings^[62]^. The inhibition of this pathway could be explored in second-line therapy to overcome this resistance in patients with OS. Now, a new drug is being tested in an ongoing phase 2 trial (identifier: NCT04616560) for the treatment of HER2-positive patients with recurrent OS. Trastuzumab-deruxtecan is a conjugated antibody-drug composed of a humanized monoclonal antibody specifically targeting HER2 and a potent topoisomerase I inhibitor as the cytotoxic drug. Trastuzumab attaches to HER2-positive cancer cells. HER-2 receptor works as a target for trastuzumab to deliver deruxtecan into OS cells^[63] ^[Figure 2].

Other members of the HER family have been investigated in OS. A moderate-to-high expression of epidermal growth factor receptor (EGFR) is present in 50%-90% of OS samples, and the expression of EGFR is associated with a higher rate of metastasis, risk of recurrence, and resistance to chemotherapy^[64,65]^. Treatments with EGFR inhibitors, such as gefitinib, inhibited OS cell growth and sensitized EGFR-expressing cells to chemotherapy, DOX, and MTX^[65]^. The combination of gefitinib with DOX and MTX also showed a synergistic impact on cell proliferation and apoptosis^[66]^. Furthermore, the monoclonal antibody cetuximab decreased OS cell motility via PI3K/AKT/mitogen-activated protein kinase (PI3K/AKT/MAPK) signaling^[67]^. Canertinib (CI-1033), EGFR, and HER2 inhibitor induced apoptosis and decreased EGFR and HER2 phosphorylation in OS cells^[68]^. ZD6474, a dual tyrosine kinase inhibitor (TKI) of EGFR and vascular endothelial growth factor receptor (VEGFR), inhibited OS cell growth, induced cell cycle arrest, and promoted apoptosis and tumor growth in nude mice^[69]^. HER4 expression is associated with low probabilities of survival and metastasis-free survival. Knockdown of HER4 decreased cell viability upon treatment with MTX and DOX and increased apoptosis of OS cells based on cleaved PARP, suggesting that downregulation of HER4 increases the sensitivity of OS cells to chemotherapeutic drugs; HER4 also interacts with NDGR1 (N-myc downstream regulated gene), which contributes to cell growth and survival in OS cells^[70]^. Afatinib is a TKI that selectively blocks the signaling of homodimers and heterodimers formed by EGFR, HER2, HER3, and HER4 in OS cell lines^[71]^. It has been observed that afatinib inhibits the proliferation, migration, and invasion of non-metastatic and metastatic OS cell lines. Moreover, it decreases the phosphorylation of HER2/EGFR receptors and downstream molecules AKT and extracellular signal-regulated kinase 1/2 (ERK1/2)^[72]^. Using sarcospheres from highly metastatic human OS cell lines, Collier *et al.* revealed that afatinib also has therapeutic potential with this technology^[73]^. Recently, a meta-analysis of the gene expression signature of primary OS samples using the Gene Expression Omnibus microarrays series was performed to establish the OS gene signature. The Characteristic Direction Signature Search Engine was used to identify the most appropriate molecules for reversing this gene expression signature in OS and propose new potential drugs. The authors found 266 genes (98 upregulated and 168 downregulated) in OS, and afatinib appeared as one of the top molecules for reversing this signature^[74]^. Afatinib is currently being tested in a phase 2 trial in pediatric tumors, including rhabdomyosarcomas and those with HER deregulation recurrent/refractory disease after receiving at least one prior standard treatment regimen (identifier: NCT02372006)^[75]^. This trial will let us see the activity of afatinib in sarcomas and patients with dysregulated HER pathway; its potential role in OS has to be evaluated directly in future clinical trials.

### PI3K, mechanistic target of rapamycin, AKT, and microtubule affinity-regulating kinase 2 pathways in OS

The PI3K/AKT signaling pathway is involved in cell survival and the RAS, RAF, and ERK/MAPK pathways, which mediate tumor proliferation and growth and are downstream of cell-surface receptors in OS^[76]^. One of the targets of this pathway is MARK2. MARK2 is a serine/threonine kinase implicated in microtubule-associated protein phosphorylation and cell cycle regulation. This protein is associated with neurological disorders, cell polarization, intracellular transport, and migration. Overexpression of MARK2 is associated with poor prognosis in patients with OS. Some mechanisms by which MARK2 may increase the resistance to cisplatin in OS have been proposed. For example, MARK2 may mediate resistance to cisplatin in OS by inhibiting apoptosis through the expression of BCL2. Another mechanism is the regulation of P-gp expression mediated by MARK2. The expression of P-gp and MARK2 in the MG-63 and MNNG/HOS OS cell lines is upregulated compared with that observed in osteoblasts. Silencing of MARK2 in OS cells also decreased P-gp expression, suggesting a relationship between these two proteins. In the case of cisplatin-resistant cells, following blockage of MARK2, P-gp expression is reduced and sensitivity to this chemotherapeutic agent is improved. This regulation may be mediated by the activation of the PI3K/AKT/nuclear factor-κB (PI3K/AKT/NF-κB) pathway^[77]^. MARK2 appears to regulate the DNA damage repair dependent on NHEJ mediated by DNA-dependent protein kinase (DNA-PK) and the catalytic subunit of the DNA-dependent protein kinase (DNA-PKcs). High levels of DNA-PKcs were correlated with poor prognosis as well as an increased risk of recurrence and metastasis in patients with OS. Furthermore, the expression of this catalytic subunit was increased in MG63 cells treated with cisplatin and etoposide^[78]^; the expression of DNA-PKcs in cisplatin-resistant MG-63 OS cells appears to be regulated by MARK2 via the PI3K/AKT/mTOR pathway. In this pathway, the protein most strongly associated with DNA damage repair is AKT; it has been reported that DNA damage activates AKT. In addition, it appears that AKT phosphorylation on S473 is dependent on DNA-PK; AKT1 forms a complex with DNA-PKcs, resulting in the activation and auto-phosphorylation of the S2056 of DNA-PKcs^[79]^. Another important protein is mTOR, a key regulator of the PI3K/AKT pathway; overactivation of mTOR is associated with resistance to cisplatin. The PI3K/AKT/mTOR and RAS/RAF/MEK/ERK pathways interact at different levels. RAS activates PI3K by interacting with its catalytic subunit, and ERK2 phosphorylates TSC complex subunit 2 (TSC2), suppressing its function and promoting the activation of mechanistic target of rapamycin complex 1 (mTORC1). Downstream of RAS/ERK is the 90 kDa ribosomal protein S6 (RPS6); this kinase phosphorylates TSC2 at Ser1798 and inactivates its tumor suppression function, allowing mTORC1 signaling^[79]^. With regard to MAPK, a special activation was demonstrated in high-grade OS specimens, showing inhibition of ERK1/2 phosphorylation and increased expression of proapoptotic proteins (e.g., BAX) that induce apoptosis in OS cells; the inhibition of ERK1/2 also increased the sensitivity of OS cells to DOX^[80]^. A phase 2 trial of sorafenib in combination with everolimus (an inhibitor of mTOR) in OS was designed to overcome the resistance to sorafenib. Sorafenib inhibits the activity of the mTORC1 complex but activates the mTORC2 complex and promotes tumor progression. Preclinical studies have shown that everolimus effectively overcomes this resistance mechanism. The study was designed to include high-grade patients with OS who had progressed after receiving a MTX/DOX/cisplatin /IFOS chemotherapy regimen that included MTX, DOX, cisplatin, and/or IFO. Interestingly, the group of patients who expressed phosphorylated-ERK1/2 (p-ERK1/2) and p-RPS6 presented a greater response to the combination of sorafenib and everolimus than the negative expression group (clinical trial identifier: NCT01804374)^[81]^. These findings suggest that, after chemotherapy, the PI3K/AKT pathway appears to be active in a subgroup of patients. Therefore, establishing the activation of the PI3K pathway prior to treatment may assist physicians in selecting patients who would benefit from treatment with an mTOR inhibitor alone or in combination with other agents. Further clinical trials based on molecular profiles are warranted to explore the combination of a mTOR inhibitor with a multikinase inhibitor with a different profile that may include the RAS/MEK/ERK pathway as targets.

## TME AND ANGIOGENESIS

OS therapy has only been partially effective, possibly due to the existence of compensatory pathways, the inherently heterogeneous nature of sarcomas, and the complex interaction with the TME. Multiple intermingled cell types, such as osteoblasts, osteoclasts, fibroblasts, immune cells, macrophages, vascular cells, mesenchymal stem cells (MSC), and hematologic progenitor cells, coexist in stroma bone^[82]^. The heterogeneity observed in different tumor cell subpopulations is modulated by different mechanisms, including the extracellular matrix (ECM) and its interactions with intracellular and extracellular elements, metabolites, oxygen tension, pH, *etc*. For instance, an alteration of the RB pathway is sufficient to induce anchorage-independent growth of these tumors; additionally, clonal evolution is dependent on the environment (hypoxia and immune infiltrate) and can result in resistance or tolerance, while microenvironment communications (e.g., angiogenesis, immune stimulation, and initiation of signal transduction) complicate this tumoral scenario^[83,84]^. Crenn *et al.* evaluated the histological response to chemotherapy (i.e., IFO, cisplatin, and DOX) in murine MOS-J cell models, mimicking various microenvironments by injecting tumor cells into subcutaneous, intramuscular paratibial, and intra-osseous sites^[85]^. A higher response to DOX was observed in the intra-osseous model compared with the intramuscular model in terms of tumor growth and necrosis, suggesting that a more vascularized intra-osseous ambient favors drug action^[25,85]^. This conclusion was based on the premise that bisphosphonates inhibit osteoclast bone resorption, reduce therapy-induced bone loss, and improve anticancer activity by inhibiting angiogenesis, invasion, tumor cell adhesion, and enhancing immunity. Additionally, in preclinical murine models, zoledronate exerted antitumor effects on OS cells, reduced tumor growth, reduced lung metastasis, and improved survival. Based on this evidence, a phase 3 trial (OS2006) evaluated the addition of zoledronate to conventional chemotherapy; unfortunately, this therapeutic strategy failed to improve the pathological preoperative response to chemotherapy and clinical outcomes. The authors explained this negative result as the effect of zoledronate on immunological parameters such as NK-cell expansion, macrophage depletion, or polarization may affect the bone microenvironment; the absence of benefit with zoledronate combined with chemotherapy may be related to a potential upregulation of RANK expression that promotes osteosarcoma pathogenesis by osteosarcoma cells^[86,25,87]^.

Other members of TME are immune cells, which include cells of the innate and non-innate immune response. Osteosarcoma has several alterations in DNA repair that translates into mutation implicated in their carcinogenesis that may produce mutational antigens attractive to immune cells; however, similar to other sarcomas, tumor mutation burden (TMB) in OS is low. Nevertheless, the microenvironment tumor-associated inflammatory infiltrate is a strong prognostic indicator of response to therapy and overall survival. Osteosarcoma had consistently low expression of PD-L1 in studies and can be classified as a “cold tumor”; however, immune cells (e.g., CD8+ cytotoxic T-lymphocytes) are associated with a favorable prognosis in OS studies.

Based on this, the phase 2 PEMBROSARC trial evaluated the anti-programmed cell death 1 (anti-PD-1) pembrolizumab in combination with alternating cyclophosphamide in 17 patients with OS; only 13.3% of patients had stable disease at 6 months^[88]^; the median progression-free survival was 1.4 months and median overall survival was 5.6 months. This study showed interesting results; for example, none of the three patients with tumor shrinkage had an expression of PD-L1 on sarcoma or immune cells and only 12% of the cases were PD-L1 positive in this study^[88]^. One explanation could be the implication of other mechanisms implicated in immune response in OS, such as other members of TME and the tumor survival mechanism^[89]^. In the PEMBROSARC trial, the authors observed an increase in the kynurenine to tryptophan ratio in the third cycle of pembrolizumab compared to the first cycle. The kynurenine pathway requires tryptophan and IDO. The authors of this trial mentioned the inhibition of IDO in combination with pembrolizumab as a strategy to be explored in these tumors^[90]^. Tumor-derived exosomes can inhibit T cell and NK cell activity through different pathways, and T cell apoptosis favors immune surveillance escape and activation of bone marrow-derived suppressor cells (MDSC). Around 36% of patients with breast cancer have exosomes containing indolamine deoxygenase (IDO), which regulates antitumor immunity by depletion of tryptophan levels, promoting inflammatory microenvironment and angiogenesis^[91,92]^. As mentioned above, it can be related to immune checkpoint inhibitor response. Release of exosomes with soluble major histocompatibility complex I chain-related proteins (SMIC) and NKG2D (natural killer cells receptor) soluble receptors are implicated in the downregulation of NK cells and cytotoxic T lymphocytes^[91,93]^. Another possible pathway implicated in OS immune response is transforming growth factor-beta (TGF-β), which plays an important role in excluding T cells from the tumor microenvironment and, unfortunately, is present in high levels in OS patients, especially in the metastatic OS setting; thus, the inhibition of this pathway may improve the antitumor effects of immunotherapy^[91,94,95]^. Combination therapy has emerged as a future strategy to enhance chemotherapy and immunotherapy; for example, in OS cells, DOX increases apoptosis in CD8+ T cells. However, this effect was reversed by the anti-PD-L1 antibody and the combination of the anti-PD-1 antibody and cisplatin inhibits tumor growth^[70]^.

This finding indicates that exploration of other pathways in the TME for the treatment of OS is warranted. Tumor-associated macrophages play a pivotal role in the regulation of local immunity, angiogenesis, and tumor cell migration; they are divided into two principal macrophage populations (M1 and M2). In OS, M1 and M2 infiltrations are associated with better and worse outcomes, respectively. It has been observed that patients with infiltration of CD68+ cells in OS tissues typically exhibit a poor response to neoadjuvant chemotherapy; however, after treatment with chemotherapy, macrophages secrete interleukin-1β (IL-1β) and reduce the sensitivity of OS to cisplatin^[96]^. Macrophages promote angiogenesis and contribute to the development of resistance to chemotherapy; the deletion of vascular endothelial growth factor A (VEGFA) in macrophages leads to normalized vascular growth, reduces hypoxia, and increases sensitivity to cisplatin^[97,98]^. Apatinib, a selective tyrosine kinase inhibitor to VEGFR2, inhibits epithelial-mesenchymal transition (EMT) and PD-L1 expression by targeting STAT3 *in vitro* and *in vivo*^[99]^. Future strategies, such as the combination of immunotherapy, chemotherapy, and anti-angiogenesis therapy or more complex alternatives such as vaccines or modified immune cells, are being tested in osteosarcoma to overcome immune surveillance escape and chemotherapy resistance^[91]^.

The ECM constitutes a three-dimensional acellular network of macromolecules that provide structural and biochemical support to cells, including malignant cells^[100]^. Moreover, the ECM is implicated in cell communication, migration, adhesion, proliferation, and differentiation. Components of the ECM (e.g., collagen, fibronectin, laminin, and proteoglycan) are implicated in OS cell growth, proliferation adhesion, invasion, metastasis, resistance to chemotherapy, and angiogenesis. A high expression of collagens, collagen triple helix repeat containing 1 (CTHRC1), and collagen type I alpha 1 chain (COL1A1) has been associated with shorter survival. Overexpression of collagen type III alpha 1 chain (COL3A1) may decrease apoptosis and promote resistance to MTX in OS cell lines^[101]^. Tumstatin is a 28 kDa protein fragment of COL4A3 (a non-collagenous domain of the alpha 3 chain in collagen IV) with an anti-angiogenic capacity that inhibits cell proliferation and induces apoptosis in OS cells^[102-104]^. Endostatin is a 20 kDa terminal-C fragment of collagen XVIII that inhibits angiogenesis by directly binding to both VEGFR1 and VGFR2. Endostatin is also associated with different surface integrins; it competes with the fibronectin pro-angiogenic ligand for binding to integrin a5β1 to disrupt cell migration, activates SRC and caveolin 1 (CAV1), disassembles focal adhesion fibers and actin stress fiber, and inhibits cell migration^[101,105]^. Both tumstatin and endostatin inhibit the phosphorylation of focal adhesion kinase (FAK) downstream of FAK; subsequently, tumstatin FAK inhibits the PI3K/AKT/mTOR/4EBP1 pathway downstream, resulting in the inhibition of endothelial protein synthesis. Endostatin inhibits the activation of the ERK1/p38 MAPK pathway that inhibits the migration of endothelial cells^[75,106]^. In preclinical models, the combination of recombinant human-endostatin (rh-endostatin) with DOX produced an important synergistic antitumor activity^[107]^. Rh-endostatin has been investigated in combination with conventional chemotherapy (i.e., MTX, DOX, and cisplatin) in patients recently diagnosed with OS; this strategy improved the clinical outcomes of chemotherapy, prolonging the 2- and 5-year event-free survival of 81% and 75% of patients, respectively, in the rh-endostatin group *vs.* 67% and 57% of patients, respectively, in the chemotherapy alone group; the relative risk in the rh-endostatin group was 0.49 (95%CI: 0.36-0.078, *P* = 0.010). Rh-endostatin reduced the 2- and 5-year distant metastasis-free survival in 82% and 79% of patients, respectively, in the combination group compared with 71% and 61% of patients, respectively, in the chemotherapy alone group; the relative risk of distant metastasis in the rh-endostatin group was 0.48 (95%CI: 0.30-0.76, *P* = 0.014)^[108,109]^. Interestingly, patients in the control group exhibited increased VEGF expression and microvascular density (MVD) after exposure to chemotherapy alone; however, patients in the rh-endostatin group showed reduced VEGF expression and MVD. Similar results have been observed in the neoadjuvant setting with DOX, cisplatin, MTX, and IFO, where rh-endostatin reduced VEGF expression and MVD, and improved distant metastasis-free survival and overall survival; these findings suggest again a protective effect in preventing lung metastasis^[110]^. This supports the rationale that early intervention with anti-angiogenic therapy in combination with conventional chemotherapy may reduce the risk of angiogenesis-dependent metastasis. Endostatin also improved clinical outcomes in patients with stage IV OS; in combination with chemotherapy, endostatin increased the progression-free survival (8.6 *vs*. 4.4 months) and the clinical benefit response (47.8% *vs*. 16.7%)^[111]^.

Angiogenesis plays an important role in the development and progression of OS; tumor micro-vessel density and VEGF expression have been associated with the prognosis of OS^[112,113]^. Chemokines promote angiogenesis in OS cells mainly through the following two pathways^[113]^. Firstly, C-C motif chemokine ligand 3 (CCL3) enhances VGEFA expression, facilitates progenitor cell migration, and promotes tube formation by downregulating the expression of microRNA (miRNA) 374b via JUN N-terminal kinase/ERK (JNK/ERK) and p38^[114]^. Secondly, CCL5 increases VEGF expression and promotes its pathway by the protein kinase C/cellular-SRC/hypoxia-inducible factor 1 alpha (PKC*δ*/c-SRC/HIF-1*α*) signaling pathway^[115]^. Higher expression of VEGFA is present in OS cells resistant to anoikis via the SRC, JNK, and ERK pathways. The use of a SRC inhibitor reduced the expression of VEGFA and angiogenesis via the inhibition of JNK and ERK activity. The overexpression of p-SRC and VEGFA is also correlated with metastatic potential in human tissues^[116]^. Relaxin is a peptide family belonging to the insulin superfamily that promotes the tumor growth, invasion, and angiogenesis of Sao-2 cells via AKT/VEGF. Relaxin H2 (RLN2) confers migratory and invasive capabilities, as well as resistance to cisplatin by modeling the AKT/NF-κB in U2OS and MG63 cells^[117]^. In patients with OS, genes of the VEGF pathway are amplified; the most frequent copy-number aberration is the amplification at 6p12-21 that involves *VEGFA *(27%), and a subset of tumors had amplifications in 4q11-12, including platelet-derived growth factor receptor A (*PDGFRA*) and kinase insert domain receptor (KDR) (18%). These findings suggest angiogenesis as a target in OS^[46]^. The activation of this pathway in OS affects survival outcomes. Notably, the receptors of the VEGFR pathway, VEGFR2 and VEGFR3, are associated with worse survival in patients with OS, while the ligand VEGFB is associated with poor histologic response to chemotherapy^[118]^. High expression of VEGFR3 and PDGFRB is associated with high-grade OS tumors^[112,118]^. Recently, a meta-analysis showed that high levels of VEGF are also associated with advanced tumor stage and metastasis, with negative consequences in terms of survival; high VEGF expression is implicated in worse disease-free survival (odds ratio = 0.25, 95%CI: 0.11-0.58, *P* = 0.001) and overall survival (odds ratio = 0.22, 95%CI: 0.13-0.35, *P* ≤ 0.001)^[119]^. These new pathways described in OS lead to the evaluation of different multikinase drugs in preclinical and clinical studies. Monotherapy with multikinase drugs has been explored in patients with OS who previously received chemotherapy. Table 1 presents the principal targeted therapies involved in signaling pathways in osteosarcoma, principally the angiogenic pathway, studied in phase 2 and observational trials as second-line therapy for OS. 

**Table 1:** Principal targeted therapies involved in the signaling pathways in osteosarcoma

**Agent**	**Signaling pathway**	**Pediatric dose**	**Adult dose**	**RR%**	**4-month PFS (%) (95%CI)**	**6-month PFS (%) (95%CI)**	**Median PFS (months) (95%CI)**	**Reference**
*Phase 2 studies*								
Apatinib*^a^*	RET, VEGFR1,2	500 mg/day^b^	750 mg/day^b^	43	57 (39-71)	37 (21-52)	4.5 (3.5-6.3)	[120]
Cabozantinib	KIT, MET, RET, VEGFR1,2,3	40 mg/m^2^/day	60 mg/day	12	71 (55-83)	52 (36-66)	6.7 (5.4-7.9)	[121]
Lenvatinib	RET, VEGFR1,2,3	14 mg/m^2^/day	14 mg/m^2^/day	7	33		3.4 (NR)	[122]
Regorafenib	KIT, RET, PDGFRB, VEGFR1,2,3	160 mg/day^c^	160 mg/day^c^	8	44.4	45	3.6 (2-7.6)	[123,124]
Sorafenib	KIT, RET, VEGFR1,2,3, PDGFRA,B	400 mg b.i.d.	400 mg b.i.d.	29	46 (28-63)	9	4 (2-5)	[125]
*Combinations*								
Sorafenib/Everolimus	PI3K/AKT, mTORC1,2		S: 400-600 mg/day E: 2.5-5 mg/day		45			[81]
*Observational studies*								
Pazopanib	VEGFR, PDGFR, KIT, FGFR		400-800 mg/day	68			5.5^d^ (2.7-7.7)	[126]

Adapted from Just *et al.*^[127]^. AKT: Protein kinase B; b.i.d.: twice daily; BSA: body surface area; CI: confidence interval; E: everolimus; FGFR: fibroblast growth factor receptor; KIT: stem cell factor receptor; mTORC: mammalian target of rapamycin complex; NR: not reported; PDGFR: platelet-derived growth factor receptor; PFS: progression-free survival; PI3K: phosphoinositide 3-kinase; RET: rearranged during transfection; RR: response rate = complete + partial responses; S: sorafenib; VEGFR: vascular endothelial growth factor receptor. ^a^Apatinib is not approved by the US Food and Drug Administration. ^b^Dose for patients with BSA < 1.5: 500 mg/day; dose for patients with BSA > 1.5: 750 mg/day. ^c^Drug is administered daily for 21 days in 28-day cycles. ^d^Study reported a 10-week PFS.

### FAK and SRC inhibitors to overcome resistance to chemotherapy

**Figure 3:**
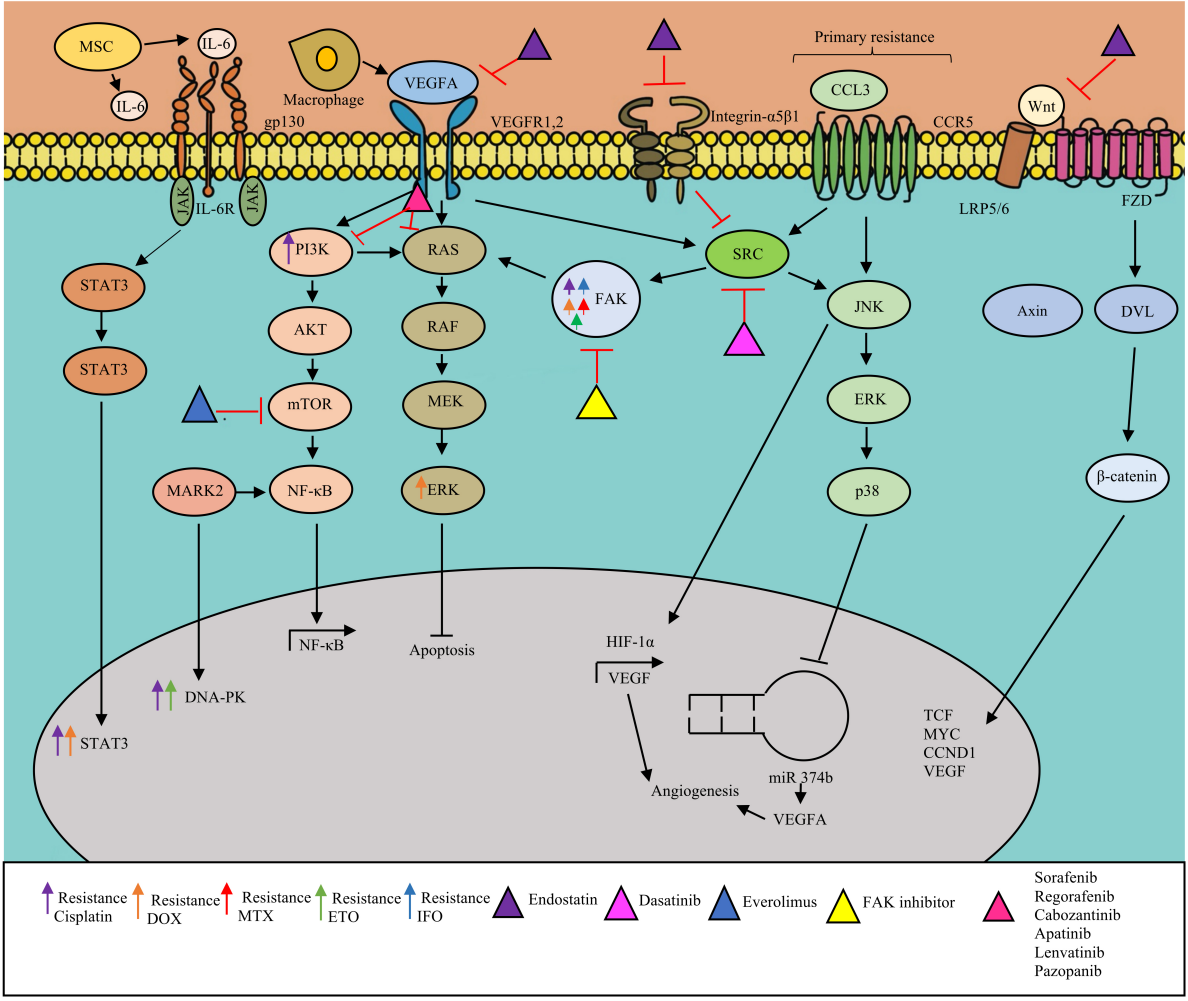
Angiogenesis and osteosarcoma microenvironment. (A) Mesenchymal stem cells (MSCs) produce IL-6 and STAT3 pathway activation promoting cisplatin and DOX resistance. (B) Macrophages promotes angiogenesis by VEGFA production in osteosarcoma. (C) VEGFA ligand binds to VEGFR activating angiogenesis pathway, throw PI3K/AKT/mTOR or Ras. Some anti-angiogenesis tyrosine kinase inhibitors alone or in combination with mTOR inhibitors are approved in second line in osteosarcoma. (D) Integrin pathway actives SRC and FAK promoting angiogenesis and apoptosis inhibition. (E) Chemokine (C-C motif) ligand 3 (CCL3) binding to G-protein coupled C-C chemokine receptor 5(CCR5) promoting VEGFA expression by downregulation of miR-374b, activation of JNK/ERK/p38 and hypoxia-inducible factor (HIF) in human osteosarcoma cells. (F) The canonical Wnt/β- catenin pathway contributes to chemotherapy resistance and osteosarcoma progression. WNT actives the Frizzled (FZD) and low-density lipoprotein receptor 5/6 (LRP5/6) binding disheveled (DVL) and Axin protein complex release of β-catenin and lead the translocation of β-catenin to the nucleus to activates genes active in chemoresistance. (G) Endostatin inhibit the activity of integrin, VEGFR and WNT pathways. AKT: Protein kinase B; CCD1: CyclinD1; DOX: doxorubicin; DNA-PK: DNA-dependent protein kinase, ERK: extracellular signal-regulated kinase; FAK: focal adhesion kinase; gp130: glycoprotein 130; mTOR: mammalian target of rapamycin; PI3K: phosphoinositide 3-kinase; IL-: interleukine-6; IL-6R: IL-6 receptor; JAK: Janus Kinase; JNK: JUN N-terminal kinase; MARK2: microtubule affinity-regulating kinase 2; MYC: myc proto-oncogene; NF-κB: nuclear factor-κB; SRC: SRC protein kinase; STAT3: signal transducer and activator of transcription 3; VEGF: vascular epidermal growth factor; VEGFR: vascular epidermal growth factor receptor.

The signaling of integrins includes SRC AKT-ERK and FAK (a non-receptor cytoplasmic protein tyrosine kinase)^[128]^. The inhibition of SRC in murine models inhibited tumor growth and decreased the metastatic potential of OS cells. Moreover, this inhibition overcame the resistance to DOX and induced apoptosis in chondrosarcoma cells^[129,130]^. This approach has been evaluated in OS in the clinical setting; monotherapy with saracatinib (AZD530) was well tolerated in patients with OS, showing a median progression-free survival of 19.4 months *vs.* 8.6 months in the placebo group (*P* = 0.47). The investigators concluded that the results of SRC inhibition alone are insufficient to suppress metastatic progression^[131]^. Dasatinib combined with ceritinib, an off-target inhibitor of insulin-like growth factor 1 receptor (IGF1R), was tested in one patient; the treatment was well tolerated and showed limited toxicity. Additionally, patient tissue analysis revealed high necrosis and extensive infiltration of macrophages, suggesting that this combination is a promising strategy^[132]^. FAK is downstream of SRC, and tyrosine 397 is the major site of autophosphorylation in the FAK catalytic domain. FAK is related to several tumor processes, such as vascular and microenvironment regulation, proliferation, motility, invasion, and survival^[133]^. Integrin-β1 (ITGβ1) activates FAK and ERK in MG63 OS cells, and plays an important role in the proliferation and differentiation process of osteoblasts^[134]^. High expression levels of FAK are associated with advanced disease and recurrence in patients with OS, rendering it a potential biomarker^[135,136]^. The levels of total FAK and p-FAK-Y397 have been evaluated in OS tissues. Overexpression of FAK was detected in OS, and overexpression of thep-FAK-Y387 was correlated with poor histologic response to chemotherapy (i.e., MTX, DOX, cisplatin, etoposide, cyclophosphamide, IFO, and carboplatin)^[135]^. In xenograft models, the decrease in FAK expression using FAK inhibitors impaired OS cell proliferation and colony formation and reduced tumor growth^[136]^. Platinum-resistant tumorspheres can acquire a dependence on FAK for growth, and the combination of a FAK inhibitor with platinum overcomes resistance to cisplatin^[137]^. Ongoing clinical trials evaluate the safety and efficacy of FAK inhibitors in different solid tumors^[138-145]^. Figure 3 shows the signal pathways involved in OS.

### TME hypoxic condition: the roles of acidosis, lactate, and adenosine in OS and therapy resistance

Angiogenesis implies newly formed microvessels with altered morphology compared with normal vessels. These abnormalities work as a biophysical barrier to the delivery of oxygen, nutriments, and antitumor therapies to a solid tumor. Oxygen delivery difficulties favor metabolic changes in TME characterized by hypoxic conditions, extracellular acidosis, substantial elevated adenosine and lactate concentrations, and nutrient deprivation^[146]^. TME hypoxic condition contributes to genetic instability, intratumorally heterogeneity, malignant progression, tumor stem cell maintenance, angiogenesis, development of treatment resistance, and metabolic reprogramming dependent on HIF-1α phenotype. These stress conditions that activate HIF-1α may serve as major drivers for recruitment, activation, polarization, and expansion of immune-suppressive stromal cells and affect the antitumor activity of the innate and adaptive immune system, as well as cancer immunotherapy. Hypoxia-/HIF-driven factors include generation and accumulation in the extracellular space of adenosine, extracellular acidosis, and favor overexpression of VEGF and activation of VEGFR related to immune suppression and anti-angiogenic therapy response^[146,147]^. Metabolic reprograming induced by hypoxia/ HIF-1α is characterized by glycolytic enzyme lactate dehydrogenase A (LDH-A) and accelerated glycolysis (Warburg effect) that contribute to lactate accumulation, affecting tumor T cell infiltration and cytokine productions, inhibits the NK and CD8+T cells cytotoxic activity and favors MDSCs infiltration. Tumor extracellular acidosis depends on: (A) the Warburg effect or upregulation/acceleration of glycolysis characterized by an intensive conversion of glucose to lactic acid and insufficient adenosine triphosphate (ATP) production to favor very fast energy supply; (B) increase in glutaminolysis; (C) ketogenesis; (D) increased ATP-hydrolysis, i.e., hydration of CO_2_-derived from oxidative metabolism and pentose phosphate pathway; and (F) bicarbonate depletion in TME^[146]^. Acidosis (extracellular pH 6.8) has immune-suppressive actions such as inhibition of the proliferative and cytotoxic activity of NK and CD8+ T cells, secretion of IFN-, and reducing the expression of T cell receptors^[148,149]^. Acid tumor microenvironment may be related to resistance in osteosarcoma. Extracellular pH in P-gp-negative cell lines reduced sensibility to DOX, and the combination of DOX with the proton pump inhibitor omeprazole enhanced its cytotoxicity capacity and reduced tumor volume in OS animal models; similarly, the pH gradient rendered in OS cells increased response to cisplatin and MTX^[150,151]^. Hypoxic stress induces cancer cells’ ATP to release through pannexin 1 PANX-1 channels and exocytosis of adenosine and promotes the accumulation of adenosine in the extracellular space of hypoxic tumors. Adenosine attenuates the activity of T cells, NK cells, and dendritic cells and enhances the suppressive capacity of T regulatory cells (Tregs) and MDSCs. Hypoxia increases the accumulation of extracellular adenosine mainly produced by enzymatic ATP catabolism; adenosine induces the expression of adenosine receptors in tumor cells, promoting growth, survival, and metastasis^[152]^. Non-regulated release of adenosine occurs from dying and damaged cells, whereas the active release involves exocytotic granules, plasma membrane-derived macrovesicles, specific ATP-binding cassette (ABC) transporters and membrane channels (connexin hemichannels and PANX1), calcium homeostasis modulator 1 (CALHM1), volume-regulated anion channels (VRACs), and maxi-anion channels (MACs). The extracellular ATP (eATP) activity is via P2 purinergic receptors, P2X7R^[153]^. TME extracellular ATP is degraded by different ectonucleotidases, principally CD39 and CD73: the ectonucleoside triphosphate diphosphohydrolase CD39 hydrolyzes ATP to ADP and AMP, while the ecto-5’-nucleotidase (CD73) hydrolyzes AMP to adenosine. CD39 is expressed in dendritic cells, tumor-infiltrating Th17 lymphocytes, and M2 macrophages. CD73 is expressed in lymphocytes T and B, stromal cells, and dendritic cells^[152,153]^; it is expressed in Treg and in higher levels on anergic CD4+ T cells, preserving self-tolerance in healthy individuals. CD73 has a role as an immune-inhibitory checkpoint molecule, contributing to tumor infiltration of regulatory immune cells such as Treg, MDSCs, or DCs, favoring an immunosuppressive microenvironment^[154]^. CD73 is overexpressed in many tumors and promotes cell migration, invasion, and chemotherapy resistance^[155]^. In human osteosarcoma cell lines, miR-16 indirectly downregulates CD73 expression and inhibits the expression of transcription factors SMAD3 and SMAD4, both implicated in CD73 expression^[154,156]^. In hypoxic TME, hypoxia induces CD73 expression via HIF-1α regulating EMT and promotes lung metastasis in triple-negative breast cancer^[157]^. Different combination strategies of CD73 pharmacological inhibition with A2BR antagonist, chemotherapy and radiotherapy, anti-PD1/PD-L1 therapy, and anti-cytotoxic T-lymphocyte antigen 4 (CTLA-4) improve cancer therapies^[152]^; Durvalumab (monoclonal antibody anti-PD-L1) and oleclumab (anti-CD73 monoclonal antibody) are being tested in NCT04668300 trial; this study includes osteosarcoma patients. The CD73 activity is mediated by P1 purinergic receptors (P1Rs), G-protein-coupled receptors divided into 4 subtypes: A1R, A2AR, A2BR, and A3R^[158]^. A1 receptor is involved in proinflammatory and anti-inflammatory processes, especially in the neurological system. A2AR protects host tissue from destruction secondary to an over-reaction of the immune response. A2AR activation inhibits DC4+ and CD8+ T-cell function and selectively inhibits proinflammatory cytokine expression, promoting the upregulation of PD-1. CTLA-4 promotes T-cell tolerance and prevents the development of IL-17, promoting the development of Foxp3+ and LAG3+ regulatory T-cells. Adenosine acting via A2AR inhibits dendritic cell function. Since chemotherapy and radiation increase eATP, a concomitant administration of A2AR antagonists during chemotherapy or radiation might lead to the expansion of tumor T cells and prevent Treg cell induction. These drugs are being explored in a clinical trial with anti-PD1-PD-L1 therapy^[159]^. A2BR is related to pathophysiological conditions associated with adenosine releases, such as ischemia and tumor hypoxia. A2BR regulates several functions including vascular tone, cytokine release, and angiogenesis. The A2B receptor promotes mesenchymal stem cell differentiation to osteoblasts and bone formation *in vivo*^[160]^. ATP release channels are also involved in osteosarcoma. Connexin 43 (Cx43), an implicated gap junction-mediated intercellular communication, is involved in proliferation suppression of human osteosarcoma U2OS cells by inhibition of the cell cycle transition, attributed to significant accumulation of hypophosphorylated RB protein that secondarily decreases kinase activities of CDK2 and -4. Cx43 seems to inhibit U2OS cells by increasing the levels of p27 protein via post-transcriptional regulatory mechanisms^[161]^. Cx43 is regulated by small ubiquitin-like modifiers (SUMOs); SUMO-conjugating enzyme UBc9 protein is overexpressed in osteosarcoma. Silencing UBc9 by siRNA inhibits osteosarcoma cell proliferation^[162]^. ALMB-0168, a humanized monoclonal antibody that specifically binds to the extracellular domain of Cx43 and activates the Cx43 hemichannels in osteocytes, enhances the activation of Cx43 hemichannels in both cultured osteocytes and mice osteocytes and promotes ATP release. ALMB-0168 reduces bone cancer growth in murine models WT Cx43; this drug also increases levels of cytotoxic lymphocytes (CD3/CD8+) and helpers (CD3/CD4+), increases the survival rate, and reduces tumor metastasis^[163]^. Another adenosine receptor, P2X7, is highly expressed in osteosarcoma tissues. The OSc receptor promotes the growth and metastasis of human HOS/MNNG cells via PI2K7AKTGSK3β/β and mTOR/HIF/VEGF signaling. Nevertheless, eATP increases plasma membrane permeability for cytotoxic molecules such as doxorubicin by opening P2X7R pores^[164]^. P2X7RA and -B are present in osteosarcoma tissue, and P2X7RB positive tumors show increased cell density depending on TME^[165]^. Shock wave-induced ATP release forms osteosarcoma U2OS cells and promotes cellular uptake and cytotoxicity of methotrexate by altering cell membrane permeability in a P2X7 receptor-dependent manner^[166]^. Adenosine, its receptors, eATP, and its transport channels are implicated in diverse pathways of osteosarcoma carcinogenesis, which makes their use attractive for managing this disease and reversing resistance mechanisms.

### Nanoparticles

The tumor microenvironment is pivotal to drug delivery in solid tumors; the different components of TME act as barriers, limit drug accumulation, and induce drug resistance. Nanoparticles are a novel tool against cancer; they accumulate passively within solid tumors via pores and fenestration of tumoral blood vessels, reaching the tumoral zone. Then, they are able to penetrate deeper into the solid tumor, where they could have high therapeutic efficacy, facing elevated interstitial fluid pressure and denser extracellular matrices. Several strategies have emerged to overcome these difficulties: surface penetrating peptides combined with magnetic field guidance, proteolytic enzymes prior to nanocarrier treatment, polymeric nanocapsules to preserve their activity for longer times, gold NPs, liposomes micelles, and micelleplexes have been tested in osteosarcoma^[167,168]^. Villegas *et al*. designed an enzyme nanocapsule attached to the surface of mesoporous silica nanoparticles as a nanocarrier model and observed higher penetrance of the nanoparticles within 3D collagen matrices of HOS OSc^[169]^. Other proposed nanoparticles include biogenic calcium carbonate with better biocompatibility, slow biodegradability, pH-sensitivity, and osteoconductivity. Specific carriers or ligands with drugs include bisphosphonates (BP), N-(2-hydroxypropyl) methacrylamide (HPMA), and tetracycline (TC), as they have potential bone targeting and are ideal for treating metastatic cancer due to their high affinity towards hydroxyapatite (HA)^[168]^. Preclinical studies using *in vitro *and *in vivo* osteosarcoma models show efficacy using thermo-sensitive hydrogel conjugated with methotrexate and alendronate, a microparticle delivery system loaded with cerium dioxide (CeO_2_) nanoparticles and doxorubicin. The developed pH-sensitive microparticles were combined with doxorubicin, liposomes of doxorubicin, lipopolymer encapsulating CRISP/Cas9 plasmids encoding VEGFA gRNA and Cas9, and more complex nanoparticles used in gene therapy, such as micelleplexes loaded with miR-145^[168]^. In the context of eATP described above, different strategies using this technology are used to prevent fast eATP degradation including highly biocompatible and biodegradable albumin nanoparticles loaded with ATP release^[170]^, a pH-sensitive nanoplatform made up of chitosan (Cs) and mesoporous HA to deliver ATP to tumor cells^[171]^.

## miRNA-MODULATED DRUG RESISTANCE IN OS

The miRNAs are small non-coding RNAs (length: 18-25 nucleotides) that repress translation and cleave mRNA by base pairing with the 3′-untranslated region of target genes. They have the potential to regulate several critical biological processes, including the differentiation, progression, apoptosis, and proliferation of tumor cells. It has been estimated that there are up to 1000 miRNAs in the human genome. More than 30% of the human genome is regulated by miRNAs that simultaneously target multiple genes; recent differences in miRNA expression profiles detected between cancer cells and their normal counterparts revealed that miRNAs are involved in the pathogenesis of cancer^[172]^. In recent years, in-depth miRNA research has validated the involvement of miRNAs in OS drug resistance, tumor initiation, and progression These oncogenic or tumor suppressor miRNAs play a role in sensitivity to chemotherapy through several mechanisms, including DNA damage response, apoptosis evasion, autophagy induction, tumor stem cell activation, and alteration of signaling pathways. Maire *et al*. performed miRNA expression profiling for 723 human miRNAs in seven OS tumors; they identified 38 miRNAs differentially expressed by ≥ 10-fold (28 and 10 were downregulated and upregulated, respectively)^[172]^. These miRNAs are involved in intracellular signaling pathways associated with drug resistance, proliferation, and metastasis in OS, including the Notch, RAS/p21, MAPK, Wnt, and Jun/FOS pathways^[172]^. It was recently shown that the upregulated miR-124 enhances the cellular response to various DNA-damaging drugs by binding to the 3′-untranslated region of the ATM interactor (ATMIN) and PARP1 mRNAs in U2OS cells^[173,174]^.

Different miRNAs have been identified as direct targets of p53, which are closely associated with drug resistance and progression in OS. Among them, members of the highly conserved miR-34 family (miR-34a, -34b, and -34c) are important components of the p53 tumor suppressor pathway. It has been observed that the expression of these miRNAs is induced by p53 in response to DNA damage or oncogenic stress^[175]^. He *et al*. reported that the miR-34 family induced G1 arrest and apoptosis in OS cells through its targets CDK6, E2F transcription factor 3 (E2F3), cyclin E2 (CCNE2), and BCL2 in a p53-dependent manner^[176]^. Additionally, it has been observed that the loss of miR-31 is associated with defects in the p53 pathway, and the overexpression of miR-31 significantly inhibits the proliferation of OS cell lines^[177]^.

In OS cells, miR-513a-5p suppressed the expression of APE-1, rendering tumor cells radiosensitive^[178]^. The use of APE-1-targeted small interfering RNA (siRNA) (i.e., pSilenceAPE-1) sensitized OS cells and tumors xenografts to the anti-angiogenic endostatin, while miR-765 downregulated APE-1 and sensitized OS cells to cisplatin. Therefore, targeting APE1 with miRNA or siRNA may be a treatment option for overcoming drug resistance in OS^[179]^.

miR-138 is the most recently discovered miRNA involved in resistance to cisplatin, which shows lower expression in OS tissue than in normal tissue. This decrease may be related to its tumor suppressor capacity. When the levels of miR-138 are restored, there is a marked inhibition of cell proliferation and invasion, as well as increased sensitivity to cisplatin. It has been observed that this change in sensitivity can be partially abolished by overexpression of the enhancer of zeste 2 polycomb repressive complex 2 subunit (*EZH2*) gene, which can block the activity of caspase 3 (CASP3), a critical enzyme for apoptosis. Therefore, *EZH2* is the specific target gene for miR-138, and this miRNA acts as a tumor suppressor in OS by enhancing the sensitivity to cisplatin^[180]^. Recent studies revealed that miR-367 executes several functions in tumors and acts as an onco-miRNA in OS. Overexpression of miR-367 is associated with strong resistance to treatment with DOX. This effect is mediated by the decreased expression of the Kruppel-like factor 4 (KLF4), BAX, and cleaved CASP3 genes, which are related to the apoptotic process and are targets of miR-367. In fact, following the downregulation of miR-367 expression, treatment of OS cells with DOX results in apoptosis^[181]^.

Based on this evidence, Wei *et al*. investigated the role of autophagy in OS cells. They observed that treatment with DOX and cisplatin increased the levels of miR-140-5p in OS cells, which stimulates autophagy^[182]^. Therefore, upregulation of miR-140-5p inhibits cell survival and resistance to DOX and cisplatin, thereby inducing autophagy^[182]^. The other miRNA involved in the process of chemoresistance is miR-184, which was investigated in OS cell lines by Lin *et al.*^[183]^. They observed that treatment with DOX induces the time-dependent expression of miR-184 in OS cell lines. It was also observed that miR-184 reduces the number of apoptotic cells after treatment by targeting and inhibiting the BCL2-like 1 (*BCL2L1*) gene, which is involved in the apoptotic process. Therefore, the upregulation of miR-184 and suppression of *BCL2L1* (which inhibits apoptosis) increased the resistance of OS cells to DOX. This study also indicated that downregulation of the expression of miR-184 increased DOX-induced apoptosis^[183]^.

Various studies have aimed to elucidate the molecular mechanisms which confer resistance to treatments, identify miRNAs that could be biomarkers of resistance to DOX and cisplatin, and recognize potential targets for future therapies based on increased sensitivity to chemotherapy. The results indicate that miRNAs are involved in the sensitivity of OS cells to several therapeutic agents [Table 2].

**Table 2:** miRNAs regulating mechanisms of drug resistance, autophagy, cancer stem cells, and signaling pathways

**miRNA**	**Alteration**	**Target gene**	**Mechanism**	**Effect on resistance**	**Drug**	**Reference**
miR-124	Downregulation	*ATMIN* *PARP1*	DNA damage response	Increase	CPT, VP‐16, and DOX	[173]
miR-15b	Downregulation	*WEE1*			DOX	[184]
miR‐101	Downregulation	*ATG4,5*	Blockage of autophagy	Increase	DOX	[185]
miR‐22	Downregulation	*HMGB1*		Increase	DOX and cisplatin	[186,187]
miR‐30a	Downregulation	*BECN1*		Increase	DOX	[188]
miR‐199a‐5p	Downregulation	*BECN1*		Increase	Cisplatin	[189]
miR‐155	Upregulation	*ATG5*	Induction of autophagy	Increase	DOX and cisplatin	[190]
miR‐140‐5p	Upregulation	*IP3K2*		Increase	DOX and cisplatin	[182]
miR‐143	Downregulation	*ATG2B* *BCL2* *LC3-II*	Activation of autophagy and stem cells	Increase	DOX	[191]
miR‐let‐7d	Downregulation or Upregulation	*HMGA2* *Lin28B* *Nanog* *Oct3,4* *Sox2*	Induction of EMT and plastic transition of CSC	Increase	DOX, cisplatin, VP-16, paclitaxel	[192]
miR‐29b‐1	Downregulation	*CD133* *N-Myc* *Nanog* *Oct3,4* *Sox2*	Reduction of CSC	Increase	DOX, cisplatin, and VP‐16	[193]
miR‐34c	Downregulation	*NOTCH1 LEF1*	Inhibition of metastasis	Increase	DOX, cisplatin, and MTX	[194]
miR‐34b	Downregulation	*PAK1 * *MDR1*	Induction of cell apoptosis	Increase	DOX, GEM, and MTX	[195]
miR‐497	Downregulation	*VEGFA*	Inhibition of proliferation	Increase	Cisplatin	[196]
miR‐221	Upregulation	*PTEN*	Promotion of proliferation and inhibition of apoptosis	Increase	Cisplatin	[197]
miR‐146b‐5p	Upregulation	*ZNRF3*	Induction of migration and metastasis	Increase	DOX, cisplatin, and MTX	[198]
miR-488	Upregulation	*BIM *	Promotion of proliferation, reduction of apoptosis	Increase	DOX	[199]
miR-765	Downregulation	*APE-1*	Inhibition of DNA damage response	Decrease	Cisplatin	[179]
miR-21	Upregulation	*Spry1, Spry2 * *PTEN *	Inhibition of migration/proliferation	Decrease	Cisplatin	[200] [201]
miR-138	Downregulation	*EZH2 *	Inhibition of migration/proliferation	Decrease	Cisplatin	[180]
miR-140-5p	Downregulation	*IP3K2 *	Induction of cell apoptosis	Decrease	DOX and cisplatin	[182]
miR-184	Upregulation	*BCL2L1 *	Inhibition of cell apoptosis	Decrease	DOX	[183]
miR-367	Upregulation	*BAX, cleaved CASP3, KLF4*	Promotion of metastasis and EMT	Decrease	DOX	[181]

APE-1: Apurinic endonuclease; ATG2B: autophagy-related 2B protein; ATMIN: ataxia telangiectasia mutated interactor; BAX: BCL2 associated X; BCL2: B-cell lymphoma 2 protein; BCL2L1: BCL2-like 1; BECN1: beclin 1; BIM: B-cell lymphoma-like protein 11; CASP3: caspase 3; CPT: camptothecin; CD133: prominin-1; CSC: cancer stem cells; DOX: doxorubicin; EMT: epithelial-to-mesenchymal transition; EZH2: enhancer of zeste 2 polycomb repressive complex 2 subunit; GEM: gemcitabine; HMGA2: high mobility group AT-hook 2; HMGB1: high‐mobility group box 1; IP3K2: inositol 1,4,5‐trisphosphate kinase 2; KLF4: Kruppel-like factor 4; LC3-II: light chain 3 type II protein; LEF1: lymphoid enhancer‐binding factor 1; LIN28B: lin-28 homolog B; *MDR1*: multidrug resistance 1; MTX: methotrexate; Nanog: Nanog homeobox; N-myc: mycn proto-oncogene; NOTCH1: Notch receptor 1; Oct 3 and 4: octamer-binding transcription factor 3 and 4; PAK1: p21‐activated protein kinase 1; PARP1: poly(ADP‐ribose) polymerase 1; PTEN: phosphatase and tensin homolog; RAB10: Ras-related protein 10; Sox2: SRY-box transcription factor 2; Spry1 and -2: sprouty; VEGFA: vascular endothelial growth factor A; VP‐16: etoposide; ZNRF3: zinc and ring finger 3.

### Non-coding miRNA in OS resistance: circRNA and lncRNA

Within the classification of non-coding RNAs are circular RNAs (circRNA) and long non-coding RNAs (lncRNA). circRNAs are characterized by a class of non-coding RNAs with a closed covalently loop without 5’-3’ polarity, with a ring structure that gives them resistance and stability to degradation by exonucleases^[202]^. circRNAs are determined for transcription^[203]^. They can be classified according to their site of origin and splicing as intron (cell nucleus)^[204]^, exon (cytoplasm)^[203]^, or exon-intron combination^[205]^. Currently, it has been revealed that circRNA can regulate gene expression at the transcriptional and post-transcriptional level by harvesting RNA-binding proteins, acting as miRNA sponges and nuclear transcriptional regulators^[206,207,208]^. In OS, circRNA has been implicated in different processes such as proliferation, invasion, apoptosis, and chemoresistance. Zhang *et al*. reported that circ_1569 is upregulated in osteosarcoma^[209]^. Overexpression of circ_001569 in OS correlated with distant metastasis, advanced tumor stage, and poor prognosis. In U2OS and MG63 cells, circ_001569 expression was elevated and increased cell proliferation. The deletion of circ_001569 decreased the proliferative capacity of OS cells. In addition, the up-regulation of circ_ 001569 promoted resistance to cisplatin, DOX, and MTX, allowing increased cell proliferation and colony formation mediated by Wnt-β-catenin pathway activation. circ_001569 deletion in OS cells decreased the expression of p-GSK3β and β-catenin. The inhibition of Wnt/β-catenin with XAV939 inhibitor decreased resistance to cisplatin, DOX, and MTX; on the contrary, LiCl (Wnt/β-catenin agonist) increased resistance to chemotherapy^[209]^.

Previous studies have identified that overexpression of circPVT1 increases the expression of the ABCB1 gene, related to classical multidrug resistance in OS cells^[210]^. circPVT1 is derived from a long non-coding RNA region located on chromosome 8q24 within the oncogene PVT1, a cancer susceptibility locus^[211]^. Kun-Peng *et al*. demonstrated that circPVT1 overexpression in OS patients correlated with the presence of metastasis and shorter survival^[210]^. The expression of circPVT1 is related to DOX and cisplatin resistance. The downregulation of circPVT1 in OS cells decreased resistance to DOX and cisplatin in OS; it appears that circPVTQ deletion decreases resistance to DOX and cisplatin through downregulation of ABCB1 gene expression^[210]^.

lncRNAs, distinguished by having more than 200 nucleotides, do not encode proteins or their coding is limited^[212]^. lncRNAs act as competitive endogenous RNA (ceRNA) regulators of miRNA expression and are driven to downstream genes^[213]^. In addition, similar to circRNAs, their functions depend on their location^[214]^. lncRNAs are involved in chromatin restoration, epigenetic organization, RNA splicing and phase splicing^[215]^. lncRNA function is involved in transcriptional and post-transcriptional signaling at the cytoplasmic level^[216,217]^. Thus, emerging evidence shows that lncRNAs are involved in OS chemoresistance. The lncRNA HOXA transcription at the distal tip (HOTTIP) located at the 5’ end of the HOXA cluster potentiates the trimethylation of lysine 4 of histone H3 for the activation of multiple 5’Hoxa genes through the WDR5/MLL complex, which will trigger tumor progression^[218,219,220]^. Recently, in osteosarcoma, upregulated HOTTIP expression correlates with advanced clinical stage, distant metastasis, and unfavorable prognosis^[221]^. Li *et al.* demonstrated that upregulated HOTTIP in OS increased the expression of cyclin D1, CDK4, and β-catenin involved in cell cycle progression^[222]^. Increased HOTTIP expression in MG63 cells promoted S-phase cell cycle and increased cisplatin resistance, which can be reversed by XAV939 (Wnt/β-catenin inhibitor). Other lncRNAs have been implicated in OS proliferation, migration, and risk of metastasis and inhibition of apoptosis, a mechanism implicated in chemotherapy resistance, suggesting an important role of lncRNAs in OS tumorigenesis and chemotherapy resistance^[222]^. A list of the most relevant circRNAs in drug resistance is presented in Table 3 and IncRNAs in Table 4.

**Table 3:** List of circular RNAs involved in drug resistance in osteosarcoma

**circRNA**	**Alteration**	**Target gene**	**Mechanism**	**Effect on resistance**	**Drug**	**Reference**
circ_001569	Upregulation	*Wnt/β catenin *	Promotes Proliferation	Increase	Cisplatin, DOX and MTX	[209]
circPVT1	Upregulation	*ABCB1*	Promotes proliferation	Increase	DOX and cisplatin	[210]
		*miR-137/TRIAP1*	Reduce apoptosis			[223]
circ_0004674	Upregulation	*circ 0004674/miR-490-3p/ABCC2 *	Promotion of proliferation, migration, cell cycle progression, and reduction of apoptosis	Increase	DOX	[224]
		*circ 0004674/miR-1254/EGFR *				[225]
		*circ_0004674/miR-142-5p/MCL-1* *miR-342-3p/FBN1 Wnt/β-catenina*				[226]
circ_0081001	Upregulation	*miR-494-3p/ TGM2*	Promotion of proliferation, metastasis, and reduction of apoptosis	Increase	MTX	[227]
circ_0000073	Upregulation	*miR-145-5p/NRAS* *miR-151-3p/NRAS *	Promotion of proliferation, migration (invasion), metastasis, and reduction of apoptosis	Increase	MTX	[228]
circPRDM2	Upregulation	*miR-760/EZH2*	Promotion of proliferation, migration (invasion), and reduction of apoptosis	Increase	DOX	[229]
circ-CHI3L1.2	Upregulation	*miR-340-5p/LPAATβ *	Inhibit EMT, migration (invasion) and reduction of apoptosis	Increase	Cisplatin	[230]

ABCB1: ATP-binding cassette subfamily B member 1; ABCC2: ATP binding cassette subfamily C member 2; DOX: doxorubicin; EGFR: epidermal growth factor receptor; EMT: epithelial-to-mesenchymal transition; EZH2: enhancer of zeste 2 polycomb repressive complex 2 subunit; FBN1: fibrillin-1; TGM2: transglutaminase-2; LPAATβ: lysophosphatidic acid acyltransferase β; MCL-1: myeloid cell leukemia-1; MTX: methotrexate; NRAS: NRAS proto-oncogene GTPase; TRIAP1: TP53 regulated inhibitor of apoptosis 1.

**Table 4:** List of long non-coding RNAs involved in drug resistance in osteosarcoma

**lncRNA**	**Alteration**	**Target gene**	**Mechanism**	**Effect on resistance**	**Drug**	**Reference**
HOTTIP	upregulated	*Wnt/β- catenin *	Promotion of proliferation and cell cycle progression	Increase	Cisplatin	[222]
ENST00000563280 (FOXC2-AS1)	upregulated	*ABCB1 HIF1A FOXC2*	Induction of migration and metastasis	Increase	DOX	[37,231,232]
LUCAT1	upregulated	*miR-200c/ABCB1*	Promotion of proliferation and migration (invasion)	Increase	MTX	[37,233]
NR-036444 (FENDRR)	Downregulation	*ABCB1* *HIF-1α FOXC2*	Induction of migration and metastasis	Increase	DOX	[37,231]
		*ABCB1/ABCC1*	Promotion of apoptosis			[234]
LINC00161	Downregulation	*miR-645/IFIT2*	Promotion of apoptosis	Decrease	Cisplatin	[235]

ABCB1: ATP-binding cassette subfamily B member 1; ABCC1: ATP binding cassette subfamily C member 1; DOX: doxorubicin; MTX: methotrexate; FENDRR: FOXF1 adjacent non-coding developmental regulatory RNA; FOXC2-AS1: Forkhead box C2 antisense RNA 1; HIF-1α: hypoxia-inducible factor 1 alpha; IFIT2: interferon-induced protein with tetratricopeptide repeats 2; LINC00161: long intergenic non-protein coding RNA 161; LUCAT1: lung cancer-related transcript 1.

## AUTOPHAGY

Autophagy is the process through which cells protect and recycle cellular components (e.g., organelles and damaged proteins) that are degraded by autophagosomes. This process allows cells to survive under stress conditions^[18]^. Autophagy is one of the most important strategies for malignant tumors to promote survival and induce resistance to chemotherapy^[236]^. Some miRNAs regulate cytotoxic activity; therefore, these miRNAs can improve the sensitivity of OS drug-resistant cell lines to chemotherapy. Li *et al.* developed a model of OS resistant MG-63 cells^[189]^. Following treatment with cisplatin, the expression of miRNA-199a-5p was decreased, the regulatory target gene Beclin 1 (*BECN1*) negative, and the expression and proportion of LC3-II to LC3-I were increased. These findings indicate the activation of autophagy. Forced overexpression of miRNA-199a-5p in OS cells resulted in the opposite result, inhibiting autophagy, enhancing the cytotoxicity of cisplatin, and reversing the resistance to cisplatin^[189]^. Chang *et al. *found that miRNA-101 can block autophagy in OS and improve the chemosensitivity of cells to DOX *in vitro*^[185]^. The results also reveal that autophagy could be induced with a certain dose of DOX in U2OS cells, while the expression of acidic vesicular organelles and another autophagy-related protein, A2g4, was decreased after transfection with miRNA-101, thereby blocking autophagy^[185]^; this blockage increased the sensitivity of OS cells to DOX. In autophagy, miRNA-199a-3p can promote multidrug resistance by inhibiting the expression of the target gene adenylate kinase 4 (*AK4*). In this process, the reduction of miRNA-199a-3p can upregulate the activated NF-κB pathway^[237]^. Regarding autophagy, Wei *et al*. found that miRNA-140-5p was upregulated in OS cells treated with cisplatin and DOX^[182]^. This effect inhibited the inositol 1,4,5‐trisphosphate kinase 2 (*IP3K2*) gene and increased autophagy, leading to the development of drug resistance in OS^[182]^. The use of chloroquine for reversing the autophagy mechanism is currently being explored in various types of tumors. In OS, chloroquine blocks the autophagic process in cisplatin-resistant OS cells; combination with rapamycin enhances the antitumor effect of this agent^[238,239]^. Autophagy has been shown to be involved in the maintenance of OS cancer stem cell (CSC) characteristics^[240]^. Stemness in CSCs is favored by autophagy in some types of tumors, e.g., breast cancer, pancreatic ductal adenocarcinoma, colon cancer, *etc*. Osteosarcoma CD271+ cells have stem cell characteristics that seem dependent on autophagic activity, with an increased expression of essential autophagy genes such as *Beclin1*, *LC3B*, *Atg5,* and *Atg7* compared to CD271 OS cells. Autophagy confers to CD271+ OS cells several advantages including resistance to hypoxic conditions, chemotherapy resistance to cisplatin and epirubicin, and tumorigenicity compared to CD271- OS cells. Autophagy-deficient CD271+ OS cells had no remarkable difference with autophagy-deficient CD271-OS, suggesting an important contribution of autophagy to the stemness of CD271+ OS cells under stress conditions. Interestingly, the inhibition of the autophagy in CD271+ OS cells reversed chemotherapy resistance, resulting in a potential pathway to be explored in OS chemotherapy resistance^[241]^.

## CANCER STEM CELLS

Cancer stem cells are a subset of cells within the microenvironment that self-renew and aberrantly mature into OS cells. These cells are characterized by a highly active DNA repair mechanism or enhanced protection against reactive oxygen species (ROS), increased expression of markers such as CD117 and CD133, and upregulation of *MDR1* protein transport genes (e.g., ABCG2) related to resistance to chemotherapy with DOX or drug efflux pumps^[18,242]^, which correspond to a combination of intrinsic and extrinsic factors contributing to CSC-mediated resistance to chemotherapy in OS. CSCs have an effective autophagy system and a complex EMT regulator capacity that lead to adaptation to TME stress conditions such as nutritional, metabolic, and oxygen privation^[243]^. Other potential biomarkers have been reported in CSCs. Honoki *et al.* found an association of high expression of ALDH-1 (aldehyde dehydrogenase 1) with resistance to chemotherapy, as well as its metastatic potential^[244]^. Schiavone *et al*. mentioned at least 20 biomarkers expressed by stem cells in OS, some of them related to chemoresistance, such as Oct4, Nanog, Sox2, CD24, CD44, Stro-1, CD133, KLF4, CBX3, and ABCA5^[245]^. Despite recognizing the role of stem cells in this tumor, an important limitation to making them a potential therapeutic target for treatment or research is that the tumor stem cell population in OS that corresponds to a population of < 1%^[246]^. Other cells with pluripotent stem cell characteristics are MSCs, which are cells highly associated with carcinogenesis, disease progression, metastasis, and drug resistance. Funes *et al*. evidenced MSC transformation in OS related to genetic alterations, such as p53 (*TP53*) and *RB* gene deficiency^[247]^. For example, Rb pathway deficiency is sufficient to induce tumor growth and progression in hypoxic conditions and favor immune system infiltrations; both conditions may contribute to chemotherapy resistance. Mutations of p53 and Rb occur in one clone of OS cells and drive to chromosomal instability and further pro-tumoral events inherited to next-generation clones, resistant to chemotherapy^[33,248]^. Additionally, aneuploidization and genomic loss of p16/CDKN2A are common causes of the transition from MSC to OS cells, and loss of p16/CDKN2A protein is a predictor of poor response to chemotherapy and worse overall survival in OS patients^[84,249,250]^. Tu *et al*. observed that activation of the signal transducer and activator of transcription 3 (STAT3) by IL-6 regulated MSCs and induced resistance to DOX and cisplatin^[251]^. Furthermore, the expression of p-STAT3 contributed to high resistance to chemotherapy in clinical samples of OS cells^[251]^. Stem cells are involved in OS tumorigenesis and treatment response and are an important research topic in this tumor.

## CONCLUSIONS

Although the application of chemotherapeutic agents contributes greatly to the effective treatment of OS, the emergence of acquired multidrug resistance remains a serious challenge. This is particularly important when using drugs that have shown greater efficacy in these tumors (e.g., cisplatin, DOX, and MTX). Several universal mechanisms underlying acquired resistance have been discovered, including drug transport, drug metabolism, aberrant drug targets, DNA damage response, apoptosis evasion, autophagy, epithelial-to-mesenchymal transition, *etc*. These mechanisms offer new directions for future management strategies that could improve oncological outcomes in patients with OS. Anti-angiogenic TKIs show that targeted therapy can improve the prognosis of this type of cancer. Novel pan-HER pathway TKIs, such as afatinib, may be effective in treating OS. New therapies that target tumor ECM pathways and the cell cycle may be helpful in overcoming resistance to chemotherapy and targeted therapy in OS.

## DECLARATIONS

### Copyright

© The Author(s) 2022.
